# Screening/diagnosis of pediatric endocrine disorders through the artificial intelligence model in different language settings

**DOI:** 10.1007/s00431-024-05527-1

**Published:** 2024-03-19

**Authors:** Lingwen Ying, Sichen Li, Chunyang Chen, Fan Yang, Xin Li, Yao Chen, Yu Ding, Guoying Chang, Juan Li, Xiumin Wang

**Affiliations:** 1 Department of Endocrinology and Metabolism, Shanghai Children’s Medical Center, School of Medicine, Shanghai Jiao Tong University, Shanghai, 200127 China; 2 Department of Neurosurgery, Huashan Hospital, Shanghai Medical College, Fudan University, Shanghai, 200040 China; 3 National Center for Neurological Disorders, Shanghai Key Laboratory of Brain Function and Restoration and Neural Regeneration, Neurosurgical Institute of Fudan University, Shanghai Clinical Medical Center of Neurosurgery, Shanghai, 200040 China; 4 Faculty of Information Technology, Monash University, Clayton, VIC 3800 Australia

**Keywords:** ChatGPT, Artificial intelligence, Pediatric endocrine and metabolism, Physician and patients, Language mode, Screening and diagnosis

## Abstract

This study is aimed at examining the impact of ChatGPT on pediatric endocrine and metabolic conditions, particularly in the areas of screening and diagnosis, in both Chinese and English modes. A 40-question questionnaire covering the four most common pediatric endocrine and metabolic conditions was posed to ChatGPT in both Chinese and English three times each. Six pediatric endocrinologists evaluated the responses. ChatGPT performed better when responding to questions in English, with an unreliable rate of 7.5% compared to 27.5% for Chinese questions, indicating a more consistent response pattern in English. Among the reliable questions, the answers were more comprehensive and satisfactory in the English mode. We also found disparities in ChatGPT’s performance when interacting with different target groups and diseases, with improved performance for questions posed by clinicians in English and better performance for questions related to diabetes and overweight/obesity in Chinese for both clinicians and patients. Language comprehension, providing incomprehensive answers, and errors in key data were the main contributors to the low scores, according to reviewer feedback.

*Conclusion*: Despite these limitations, as ChatGPT continues to evolve and expand its network, it has significant potential as a practical and effective tool for clinical diagnosis and treatment.

**Table:** 

**What is Known:** *• The deep learning-based large-language model ChatGPT holds great promise for improving clinical practice for both physicians and patients and has the potential to increase the speed and accuracy of disease screening and diagnosis, as well as enhance the overall efficiency of the medical process. However, the reliability and appropriateness of AI model responses in specific field remains unclear.* *• This study focused on the reliability and appropriateness of AI model responses to straightforward and fundamental questions related to the four most prevalent pediatric endocrine and metabolic disorders, for both healthcare providers and patients, in different language scenarios.*	
**What is New:** *• The AI model performed better when responding to questions in English, with more consistent, as well as more comprehensive and satisfactory responses. In addition, we also found disparities in ChatGPT’s performance when interacting with different target groups and different diseases.* *• Despite these limitations, as ChatGPT continues to evolve and expand its network, it has significant potential as a practical and effective tool for clinical diagnosis and treatment.*

**Supplementary Information:**

The online version contains supplementary material available at 10.1007/s00431-024-05527-1.

## Introduction

With the ongoing advancement of intelligent technologies such as artificial intelligence (AI) and machine learning techniques, coupled with advancements in computing power, learning algorithms, and the availability of large datasets sourced from medical records and wearable health monitors, AI is playing an increasingly prominent role in medicine and the healthcare system [1–3]. For instance, AI can improve prediction accuracy, enhance service delivery, and aid in disease detection. It can be used in various aspects of healthcare, from molecular and genetic testing to medical imaging and infectious disease outbreak predictions as part of health emergency protection programs. These AI advancements provide prompt, economical, and higher-quality solutions for modern diagnosis, prevention, treatment, and healthcare breakthroughs.

ChatGPT, developed by OpenAI, is a state-of-the-art deep learning-based large-language model [4]. It has been trained on vast amounts of medical data and can generate relevant information about various diseases, symptoms, and treatments in a chat-based, answer-to-question manner. Hence, ChatGPT holds great promise for improving clinical practice for both physicians and patients. It has the potential to increase the speed and accuracy of disease screening and diagnosis, as well as enhance the overall efficiency of the medical process.

Thus, further studies are necessary to determine the reliability and appropriateness of AI model responses. Given the escalating worries of parents about their children’s growth and development, this study focused on straightforward and fundamental questions related to the four most prevalent pediatric endocrine and metabolic disorders for both healthcare providers and patients. Additionally, to examine the model’s ability to respond in different language scenarios, the questions were presented in both Chinese and English.

## Methods

This study was conducted in February 2023 to evaluate the quality of responses provided by the AI model, ChatGPT, to questions related to pediatric endocrine and metabolic conditions. The comprehensive questionnaire consisted of 40 questions and targeted both medical practitioners and patients and covered the four most common pediatric conditions: short stature, precocious/delayed puberty, overweight/obesity, and diabetes mellitus.

**Fig. 1:**
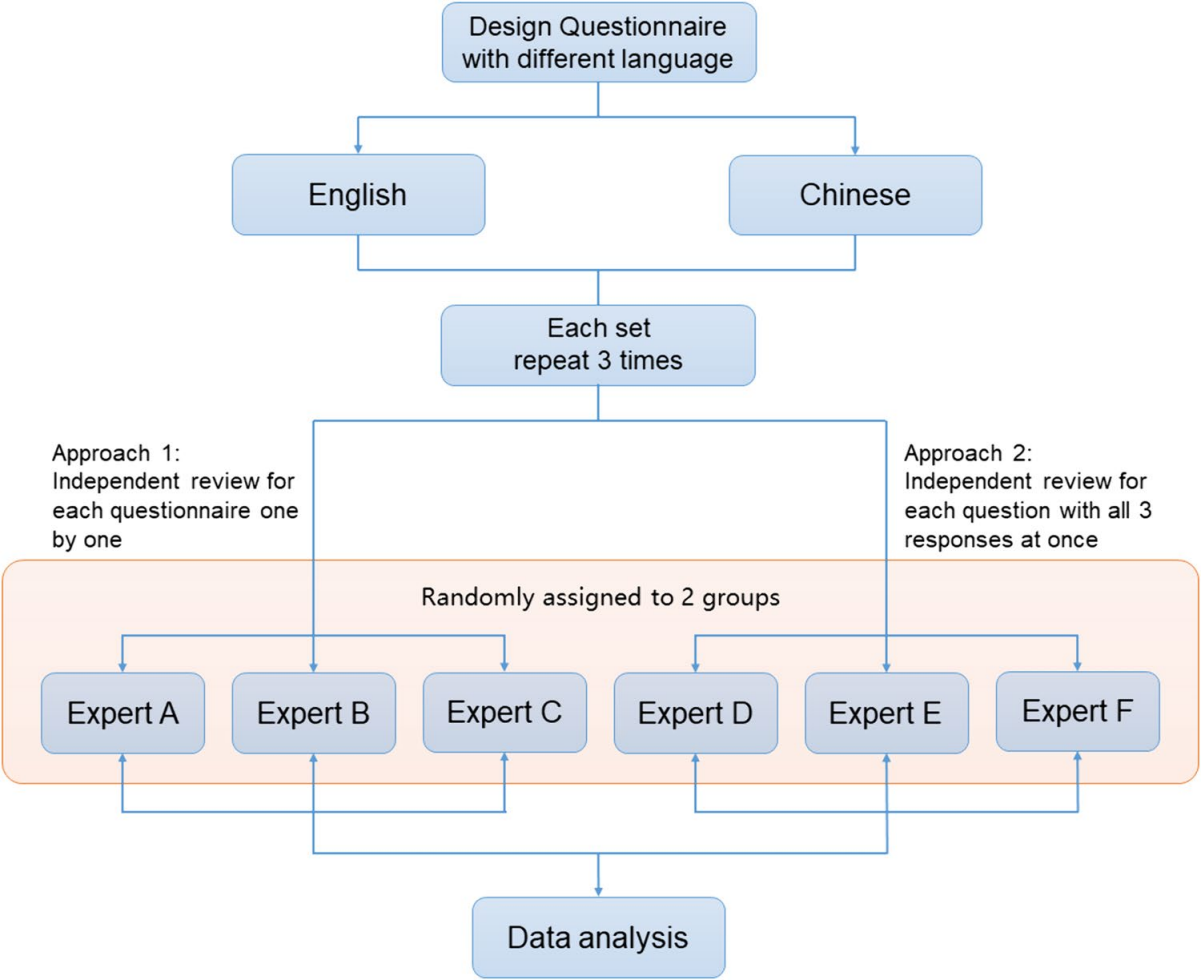
Evaluation process of AI responses to questionnaire with different languages. The questionnaire consisted of 40 questions regarding the four most prevalent pediatric endocrine and metabolic disorders and was targeted towards both medical practitioners and patients. The questions were posed in both Chinese and English and evaluated by six pediatric endocrinologists who were divided into two groups based on the marking method (Approach 1 and Approach 2). The responses were graded as unreliable, inappropriate, or appropriate, with the “appropriate” category further classified as satisfactory, good, or excellent

The questions were posed to the AI interface three times each in both Chinese and American English (totally 6 times), and the responses were recorded [5, 6]. A total of 6 experienced pediatric endocrinologist participated and were randomly divided into two groups based on the random table method (Fig. 1); then, they graded each set of three responses in both Chinese and English. In Approach 1, the reviewers were given one questionnaire with all the questions and related responses at once (like an exam paper). In Approach 2, the three responses for each question were combined and sent to the reviewers one by one (i.e., Question 1 with answers 1, 2, and 3).

This grading system evaluates responses based on three aspects: reliability, accuracy, and consistency. Responses that fail to be consistent are labeled as “unreliable”. Responses that are consistent but inaccurate are given 0 points and are labeled as “inappropriate”. On the other hand, responses that are consistent and accurate receive 1, 2, or 3 points based on their quality and are considered “appropriate”. Within this category, “satisfactory” responses receive 1 point, “good” responses receive 2 points, and “excellent” responses receive 3 points, with the score reflecting how closely the response aligns with the reviewer’s expectations.

All statistical analyses were performed using SPSS Statistics, version 24.0 (SPSS Inc., Chicago, IL). All scores of reliable responses were presented with mean ± standard error of mean (SEM). The expert evaluation results were analyzed using the Wilcoxon rank sum test and the Mann–Whitney *U* test to compare results between groups, including language (Chinese or English), disease type, and target population. Statistical significance was determined by a two-tailed *P* value of less than 0.05.

## Results

Table 1 illustrates the varying responses of the AI model across different languages. It is evident that ChatGPT performs better when responding to questions in English, with an “unreliable” rate of 7.5% (3 out of 40) compared to 27.5% (11 out of 40) for Chinese questions, which implies a more consistent response pattern in English. Furthermore, among the “reliable” questions, ChatGPT received higher scores for English questions than for Chinese questions, indicating that the answers were more comprehensive and satisfactory in the English mode.

**Table 1:** Assessment of the accuracy and reliability of an AI-powered chat platform for screening and diagnosing common pediatric endocrine and metabolic disorders by pediatric endocrinologists (English questions)

**Question**	**Reviewer grade for response for English questionnaire**		**Reviewer grade for response for Chinese questionnaire**	
	**Approach 1**	**Approach 2**	**Approach 1**	**Approach 2**
**For clinicians**				
** Short stature**				
** 1. What are the diagnostic criteria for short stature?**	2.22 ± 0.22	2.33 ± 0.17	Unreliable	
** 2. What is the pathology of short stature?**	2.67 ± 0.17	2.67 ± 0.17	2.11 ± 0.20	2.08 ± 0.26
** 3. How to choose growth hormone stimulation test?**	1.78 ± 0.22	2.78 ± 0.15^**^	Unreliable	
** 4. How to interpretate the results of growth hormone provocation test?**	2.00 ± 0.17	2.44 ± 0.18	Unreliable	
** 5. What is the normal value of children’s growth rates?**	2.00 ± 0.29	2.22 ± 0.22	Unreliable	
** 6. How to measure bone age?**	2.22 ± 0.22	3.00 ± 0.00^*^	Unreliable	
** 7. How to define retardation of bone age?**	2.00 ± 0.17	2.67 ± 0.17^*^	Unreliable	
** 8. When to perform IGF-1 test?**	Unreliable		1.22 ± 0.15	1.67 ± 0.31
** Precocious puberty/delayed puberty**				
** 1. What are the diagnostic criteria for precocious puberty?**	1.78 ± 0.15	2.56 ± 0.18^*^	0.67 ± 0.17	1.42 ± 0.19*
** 2. How to define delayed puberty?**	2.33 ± 0.24	2.33 ± 0.17	1.11 ± 0.11	1.11 ± 0.11
** 3. How to perform Gonadotropin-releasing hormone (GnRH) stimulation test?**	2.33 ± 0.24	2.89 ± 0.11	1.00 ± 0.17	2.58 ± 0.15^**^
** 4. What may cause delayed puberty?**	2.44 ± 0.24	2.89 ± 0.11	1.89 ± 0.26	2.50 ± 0.15
** 5. Why girls with delayed puberty should test for their karyotype?**	2.22 ± 0.22	2.89 ± 0.11^*^	1.56 ± 0.18	2.00 ± 0.21
** 6. Please describe secondary sexual characteristics develop during puberty**	2.33 ± 0.17	2.67 ± 0.17	1.67 ± 0.24	2.08 ± 0.08
** 7. How to distinguish true precocious puberty and pseudo precocious puberty?**	2.44 ± 0.18	2.67 ± 0.17	Unreliable	
** 8. When to perform GnRH stimulation test?**	2.33 ± 0.17	2.67 ± 0.17	1.33 ± 0.33	2.33 ± 0.23^*^
** Diabetes mellitus**				
** 1. What are the diagnostic criteria for diabetes?**	2.44 ± 0.29	2.11 ± 0.26	Unreliable	
** 2. How to perform 75 g oral glucose tolerance test (OGTT)?**	2.44 ± 0.24	2.78 ± 0.15	1.78 ± 0.28	2.33 ± 0.23
** 3. Types/classifications of diabetes**	1.89 ± 0.20	2.00 ± 0.24	1.56 ± 0.34	2.08 ± 0.26
** 4. What are the factors that affecting the levels of glycated hemoglobin A**_**1c**_**?**	2.78 ± 0.15	3.00 ± 0.00	1.56 ± 0.18	2.33 ± 0.23^*^
** 5. What is the treatment target for diabetic children?**	2.33 ± 0.17	2.44 ± 0.24	1.89 ± 0.26	2.08 ± 0.19
** 6. What may be the potential indicators for early identify diabetes for children?**	2.00 ± 0.29	2.78 ± 0.15	1.78 ± 0.28	2.00 ± 0.37
** 7. What are considered risk factors for diabetes?**	2.22 ± 0.22	2.89 ± 0.11^*^	2.00 ± 0.24	2.33 ± 0.23
** 8. When to screen for diabetes?**	2.11 ± 0.26	3.00 ± 0.00^*^	2.22 ± 0.22	2.50 ± 0.15
** Overweight/obesity**				
** 1. How to define obesity and overweight for Chinese children?**	Unreliable		1.44 ± 0.29	2.25 ± 0.22
** 2. What is the “gold standard” of obesity/overweight?**	Unreliable		Unreliable	
** 3. How to grade the degree of obesity?**	1.89 ± 0.26	3.00 ± 0.00^**^	1.89 ± 0.26	2.00 ± 0.25
** 4. What may cause obesity in children?**	2.44 ± 0.18	3.00 ± 0.00	2.22 ± 0.15	2.42 ± 0.19
** 5. What are the general symptoms of children with obesity/overweight?**	2.11 ± 0.26	2.67 ± 0.17	1.78 ± 0.28	2.25 ± 0.25
** 6. What is acanthosis nigricans?**	2.22 ± 0.15	3.00 ± 0.00^*^	1.11 ± 0.26	1.50 ± 0.15
** 7. What is insulin resistance?**	2.22 ± 0.22	3.00 ± 0.00^*^	2.11 ± 0.26	2.25 ± 0.13
** 8. Can semaglutide be used to lose weight in obese children?**	2.67 ± 0.17	3.00 ± 0.00	Unreliable	
** For patients**				
** 1. I have an 8-year-old daughter with her height 120 cm, is she too short?**	2.67 ± 0.17	2.67 ± 0.17	Unreliable	
** 2. My daughter is 14 years with her height of 157 cm. Can she use growth hormone to grow to more than 160 cm?**	2.67 ± 0.17	2.67 ± 0.17	2.11 ± 0.20	2.42 ± 0.19
** 3. My son is currently 10 years old and his voice has changed, is this normal?**	1.78 ± 0.22	2.00 ± 0.29	1.11 ± 0.11	1.50 ± 0.34
** 4. My daughter is 16 years, and she has not had her period yet. Is this normal?**	1.78 ± 0.28	2.78 ± 0.15^*^	1.00 ± 0.00	1.25 ± 0.25
** 5. Both my parents have diabetes, how likely am I to get diabetes?**	2.33 ± 0.24	2.44 ± 0.18	1.78 ± 0.22	2.25 ± 0.25
** 6. My child has been diagnosed with type 1 diabetes. What is the proper glucose target?**	2.11 ± 0.26	2.67 ± 0.17	2.00 ± 0.17	2.25 ± 0.25
** 7. How to lose weight?**	2.67 ± 0.17	3.00 ± 0.00	2.44 ± 0.18	2.75 ± 0.13
** 8. My doctor said that I'm obese, can I do bariatric surgery?**	2.22 ± 0.28	2.56 ± 0.18	2.00 ± 0.00	2.33 ± 0.19

The scores were presented as mean ± standard error of mean (SEM). ^*^P < 0.05 approach 1 vs. approach 2; ^**^P < 0.01 approach 1 vs. approach 2. Each set with all those questions was posed on the interface 3 times. Each set of 3 responses was graded in 2 approaches: (1) Approach 1: each questionnaire with all those questions and related responses above (like an exam paper) was sent to reviewers one by one; (2) Approach 2: all three responses for each question were integrated and sent to reviewers one by one (i.e., question 1 with answers 1, 2, and 3). Each set of responses was graded as appropriate, inappropriate, or unreliable, while appropriate was further classed as satisfactory (1 point), good (2 points), and excellent (3 points). Appropriate indicates that all 3 responses were internally consistent and generally similar to what the reviewer might recommend; inappropriate, all 3 responses were internally consistent but factually inaccurate and/or different from what the reviewer might recommend; and unreliable, the 3 responses were inconsistent with each other

The primary cause of the discrepancy in scores, according to reviewer feedback, is language, both in comprehending the questions and providing answers. The translation process, language habits, and grammar used in the answers, as well as technical errors such as mistaking “Bone density” for “Bone age” or “Drinking alcohol” for “Drinking the glucose solution”, play a significant role in the low scores. Additionally, the low scores can be attributed to answers that are lacking in comprehensiveness, such as the absence of a specific diagnostic threshold or clear source for relevant diagnostic criteria. Errors in key data also contribute to the low scores. For example, in the Chinese mode answer to the question “What is the normal value of children’s growth rates?”, there are inconsistencies in all three answers, including one particularly surprising answer stating: “The annual growth rate of 0–1-year-old babies is 50–70 cm, and for 2–3-year-old children it is about 25–30 cm. The growth rate of 4–5-year-olds is about 20–25 cm, and for 6–12-year-olds it is about 10–20 cm.”

Additionally, our findings suggest that varying evaluation methods can also affect the score of the AI model, as shown in Table 1. Regardless of whether the questions were in English or Chinese, when the answers to the same question were combined and presented to the reviewer through Approach 2, the final score of the questions improved somewhat, but the overall pattern of scores for each question did not show any significant changes.

**Fig. 2:**
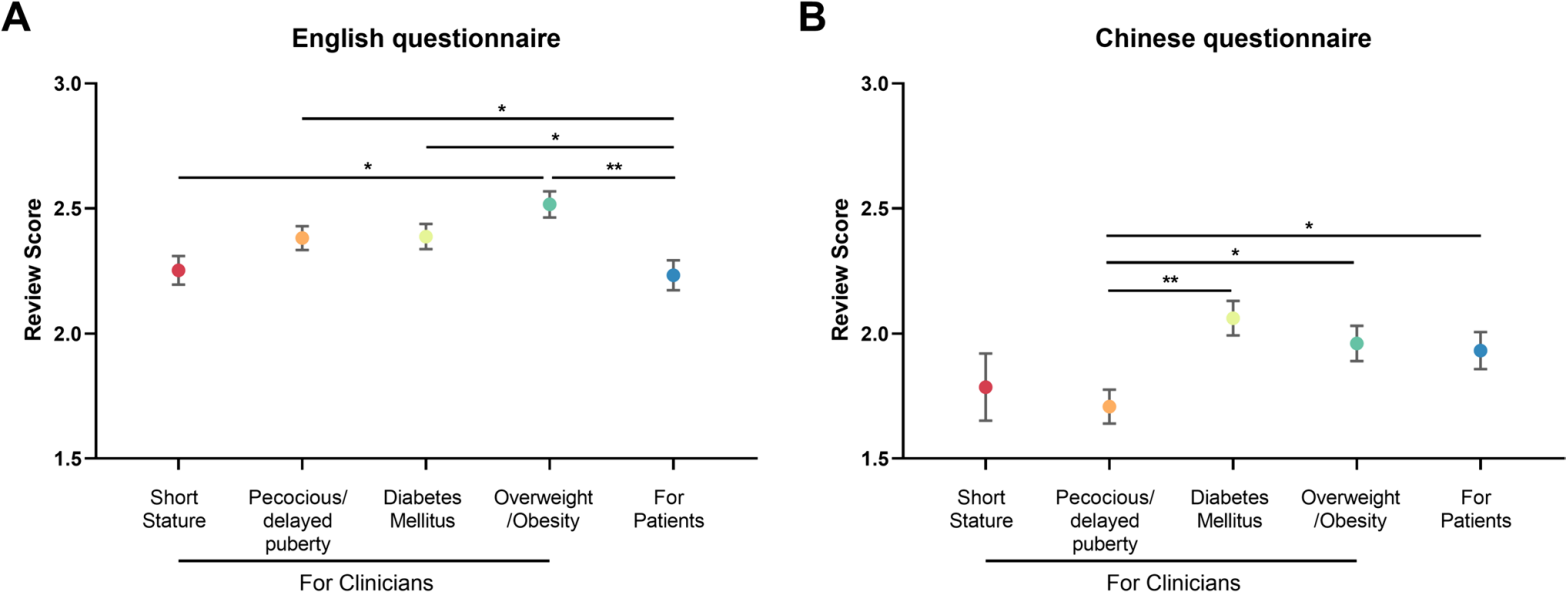
Scores of subgroups of AI models in response to English **(A)** and Chinese **(B)** questionnaires. ^*^P < 0.05; ^**^P < 0.01

Subgroup analysis was conducted to evaluate the AI model’s performance on the four most common pediatric endocrine diseases covered in the questionnaire. The results revealed that the AI model was most effective in answering questions related to diabetes mellitus and overweight/obesity. Figure 2 shows that in the English model, the AI was best able to respond to questions about overweight/obesity, followed by diabetes mellitus, precocious/delayed puberty, and performed least well in questions about short stature. In the Chinese model, most answers to questions about short stature were deemed unreliable. Among the remaining reliable answers, the AI model performed best in questions about diabetes mellitus, followed by overweight/obesity, and performed worst in questions about both short stature and precocious/delayed puberty.

Our research revealed disparities in the performance of the AI model when interacting with different target groups. In the English mode, the model demonstrated improved performance with questions posed by clinicians when compared to those posed by patients. However, for patient-related questions, except for questions about puberty, the AI model provided acceptable answers to the other six questions. In the Chinese mode, the AI model performed better in questions related to diabetes mellitus and overweight/obesity for both clinicians and patients.

## Discussion

Our study explored the reliability and appropriateness of a popular online AI model in answering simple questions related to pediatric endocrine conditions. The evaluation by pediatric endocrinologists showed that the model performed well to some degree. Our results highlight the potential of AI to augment healthcare providers’ expertise by delivering accurate medical information, diagnostic suggestions, and treatment recommendations, much like the reference tool UpToDate. The model may also be useful in triaging common queries related to pediatric endocrine diseases.

Despite progress, there are still challenges in the application of interactive AI. The primary issue is language, particularly its semantic understanding. Our findings indicate that the AI model is more consistent and generates more satisfactory answers in the English questioning system compared to Chinese. This may be because English is the native language of the system, while Chinese presents a greater challenge in terms of comprehension. Additionally, the AI model also exhibits deviations in semantic understanding. For instance, in response to the question “What may be the potential indicators for early identification of diabetes in children?”, the original intention was to inquire about suitable laboratory indicators such as advanced glycation end products (AGEs), glycated albumin (GA), and 1,5-anhydroglucitol (1,5-AG). However, when utilizing the English mode, the question was primarily classified as clinical symptoms that may indicate diabetes. Whereas in the Chinese mode, ChatGPT categorizes questions as being about high-risk factors for diabetes to potentially receive a more scientifically accurate response from ChatGPT to modify the keywords in the query by using more limited terms such as “biomarker” or “index” instead of “indicator”. Moreover, there is a considerable delay in the system’s response times (approximately 2 to 3 times longer) when inquiries are made in Chinese compared to English. Therefore, English remains the most suitable language for the interactive AI system at present, and further research is needed to determine the suitability of its responses in other languages.

Besides, in the process of asking questions, it is crucial to provide a clear and accurate description of the problem at hand. For instance, when asking about “the diagnostic criteria for diabetes”, it is important to note that these criteria are constantly evolving and that different organizations may have different criteria for different populations, such as the American Diabetes Association (ADA) or the World Health Organization (WHO) [7, 8]. Additionally, there are different diagnostic criteria for different forms of diabetes, such as gestational diabetes mellitus (GDM) and fulminant type 1 diabetes mellitus (FT1DM). However, AI models like ChatGPT may not have the capacity to fully consider these nuances. The answers ChatGPT provides may not always align with the requirements of a clinical setting and may not include references or the source of the criteria. This could pose a risk, especially for non-specialist doctors or non-medical workers who rely on AI models for information. To minimize these risks, it is essential to provide a detailed description when posing requirements, for example, “specifying the diagnostic criteria for type 2 diabetes in the 2010 ADA guidelines”. Additionally, for non-specialist physicians and other non-medical individuals (such as patients) searching for information, the AI model can help decrease the occurrence of such issues by including specific reference sources in its responses.

AI models still have limitations in their analytical abilities, particularly when it comes to issues related to short stature and precocious puberty/delayed puberty. For instance, when asked “My son is 10 years old and his voice has changed, is this normal?”, the response from ChatGPT, whether in Chinese or English, is “this is normal”. However, this is not always accurate. The progression of secondary sexual characteristics during a child’s growth and development indicates that voice change is one of the final stages of pubertal development in boys, typically occurring approximately 1.5 to 2 years after the start of puberty. Hence, a 10-year-old boy whose voice has changed is actually experiencing an early onset, and further evaluation is needed. On a positive note, ChatGPT’s answers to these types of questions consistently emphasize that “every child develops at their own pace, and some may experience changes earlier or later than others”. As a result, medical consultation is recommended.

This study examined the impact of ChatGPT on four common diseases screening and diagnosis in various linguistic contexts, to different targeted population. However, it does have some limitations. First, just six pediatric endocrinologists were involved, which may result in variations in the scoring system based on their individual knowledge, clinical experience, and desired level of detail in answers. To enhance the validity of these findings, it would be ideal to include a larger, diverse sample of reviewers in future studies, taking into account factors such as specialist vs. non-specialist status and level of experience. Second, the focus of this study is on the screening and diagnosis of common endocrine diseases, while the impact of AI models on other aspects of health care, such as prevention and public education, remains to be seen. Finally, the accuracy and reliability of AI models heavily depend on the quality and bias of the training data used. For this research, we utilized ChatGPT version 2023.1.31. However, it is important to note that the correction training of its wrong answers cannot be applied to another set. Therefore, more research is necessary to determine whether continuously updating and training ChatGPT can improve its accuracy of knowledge extraction.

Overall, ChatGPT shows great potential as a more intelligent search engine that can assist physicians in clinical practice and may contribute to the popularization and triage of related diseases. However, there are limitations to using ChatGPT in clinical practice. For example, it does not ask any questions and, therefore, cannot gather additional information about the patient’s chief complaints. Moreover, it cannot assess the accuracy of the patient’s subjective expression, nor can it perform related clinical skills like physical examination. As a result, large-language models like ChatGPT cannot replace the expertise of specialists in the diagnosis and treatment of diseases. Despite these limitations, as ChatGPT continues to develop and expand its network, it holds great potential as a practical and effective tool for clinical diagnosis and treatment.

### Supplementary Information

Below is the link to the electronic supplementary material.

Supplementary file1 (DOCX 22 KB)

## Data Availability

All of the relevant data are included in the article/supplementary materials. Further inquiries can be directed to the corresponding authors.

## Abbreviations

1,5-AG: 1,5-Anhydroglucitol
ADA: American Diabetes Association
AGEs: Advanced glycation end products
AI: Artificial intelligence
FT1DM: Fulminant type 1 diabetes mellitus
GA: Glycated albumin
GDM: Gestational diabetes mellitus
SEM: Standard error of mean
WHO: World Health Organization

## References

[1] Martinez-Millana A, Saez-Saez A, Tornero-Costa R (2022). Artificial intelligence and its impact on the domains of universal health coverage, health emergencies and health promotion: an overview of systematic reviews. Int J Med Inform.

[2] Korngiebel DM, Mooney SD (2021). Considering the possibilities and pitfalls of Generative Pre-trained Transformer 3 (GPT-3) in healthcare delivery. NPJ Digit Med.

[3] Ahuja AS (2019). The impact of artificial intelligence in medicine on the future role of the physician. PeerJ.

[4] van Dis EAM, Bollen J, Zuidema W (2023). ChatGPT: five priorities for research. Nature.

[5] ChatGPT: optimizing language models for dialogue. https://chat.openai.com/chat. Accessed on 19 Feb 2023

[6] Sarraju A, Bruemmer D, Van Iterson E (2023). Appropriateness of cardiovascular disease prevention recommendations obtained from a popular online chat-based artificial intelligence model. JAMA.

[7] American Diabetes Association (2010) Standards of medical care in diabetes--2010. Diabetes Care 33(Suppl 1):S11–61 10.2337/dc10-S011 PMC2797382 20042772

[8] Alberti KG, Zimmet PZ (1998). Definition, diagnosis and classification of diabetes mellitus and its complications. Part 1: diagnosis and classification of diabetes mellitus provisional report of a WHO consultation. Diabet Med.

