# Supplementary material for: Screening/diagnosis of pediatric endocrine disorders through the artificial intelligence model in different language settings
Source: Eur J Pediatr. 2024 Mar 19;183(6):2655–61. doi: 10.1007/s00431-024-05527-1 (PMC11098926; doi:10.1007/s00431-024-05527-1)
Supplement: Supplementary file 1 — Supplementary file1 (DOCX 22 KB) [file 431_2024_5527_MOESM1_ESM.docx]

**Supplementary Table 1. Assessment of the Accuracy and Reliability of an AI-Powered Chat Platform for Screening and Diagnosing Common Pediatric Endocrine and Metabolic Disorders by Pediatric Endocrinologists (Chinese questions)**

| Question |
| --- |
|  |
| For Clinician |
| Short stature |
| 1. 矮小症的诊断标准是什么？ |
| 2. 矮小症的发病原因。 |
| 3. 生长激素激发试验有哪些？如何选择？ |
| 4. 生长激素激发试验结果解读？ |
| 5. 儿童年身高增长速率正常值。 |
| 6. 骨龄如何测定？ |
| 7. 如何定义骨龄落后？ |
| 8. 什么情况下需考虑行IGF-1生成试验？ |
| Precocious puberty/Delayed puberty |
| 1. 性早熟的诊断标准是什么？ |
| 2. 什么是青春期延迟？ |
| 3. 如何进行促性腺激素释放激素激发试验？ |
| 4. 青春期延迟的病因。 |
| 5. 为何青春期延迟的女性需完善染色体核型？ |
| 6. 第二性征发育包括哪些？ |
| 7. 如何界定真性性早熟和假性性早熟？ |
| 8. 什么情况下需考虑行GnRH激发试验 |
| Diabetes mellitus |
| 1. 糖尿病的诊断标准是什么？ |
| 2. 如何实施75g OGTT？ |
| 3. 糖尿病的具体分型。 |
| 4. 糖化血红蛋白的影响因素是什么？ |
| 5. 儿童糖尿病的目标血糖。 |
| 6. 儿童糖尿病的潜在早期筛查指标。 |
| 7. 糖尿病的危险因素是什么？ |
| 8. 何种情况下需筛查糖尿病？ |
| Overweight/Obesity |
| 1. 中国儿童肥胖和超重的诊断标准是什么？ |
| 2. 诊断肥胖/超重的金标准是？ |
| 3. 肥胖程度的分级。 |
| 4. 引起儿童肥胖的原因。 |
| 5. 肥胖/超重儿童一般有那些体征？ |
| 6. 请定义黑棘皮病？ |
| 7. 什么是胰岛素抵抗？ |
| 8. 司美格鲁肽能否用于肥胖儿童的治疗？ |
| For patients |
| 1. 我有一个8岁的女儿，她目前身高120cm，她的身高偏矮吗？ |
| 2. 我的女儿14岁了，只有157cm，可以通过打生长激素长到160cm么？ |
| 3. 我有一个10岁的儿子，已经变声了，这正常吗？ |
| 4. 我的女儿16岁了，目前还没来例假，这正常吗？ |
| 5. 我的父母有糖尿病，我有多大可能得糖尿病？ |
| 6. 我家小孩被诊断为1型糖尿病，他的血糖应该控制在多少比较合适？ |
| 7. 怎么减重？ |
| 8. 医生说我有点胖，我可以去做减重手术么？ |

Note: Each set with all those questions was posed on the interface 3 times. Each set of 3 responses was graded in 2 approaches: 1). Approach 1: each questionnaire with all those questions and related responses above (like an exam paper) was sent to reviewers one by one; 2). Approach 2: all three responses for each question were integrated and sent to reviewers one by one (i.e., question 1 with answers 1, 2 and 3). Each set of responses was graded as appropriate, inappropriate, or unreliable, while appropriate was further classed as satisfactory (1 point), good (2 point) and excellent (3 point). Appropriate indicates that all 3 responses were internally consistent and generally similar to what the reviewer might recommend; inappropriate, all 3 responses were internally consistent but factually inaccurate and/or different from what the reviewer might recommend; and unreliable, the 3 responses were inconsistent with each other.
